# Integrated Chemical and Biochemical Treatments to Produce Protein and Microbial Lipid Food Ingredients from Ryegrass

**DOI:** 10.1021/acssuschemeng.5c02288

**Published:** 2025-06-13

**Authors:** Fatma Guler, Yubin Ding, Hannah S. Leese, Bernardo Castro-Dominguez, Christopher J. Chuck

**Affiliations:** Department of Chemical Engineering, 1555 University of Bath, Claverton Down, Bath BA2 7AY, U.K.

**Keywords:** Italian ryegrass, plant protein, biorefinery, mechanochemical treatment, medium formulation, oleaginous yeast, microbial lipid

## Abstract

Pretreatment methods play a pivotal role in the efficient breakdown of lignocellulosic biomass to produce highly digestible solids. Incorporating multistage or combined pretreatments provides increased efficiency and further carbohydrate depolymerization. Unlike other biomass feedstocks, ryegrass is a promising nutritional plant protein source with a protein content of 6–16%. In this study, protein extraction was combined with a mechanochemical pretreatment and biochemical fermentation stage to maximize the value of products from the system. To this end, the effectiveness of cold press, ball mill, and mild alkaline combined treatments on protein extraction was investigated, and the efficiency of enzymatic saccharification of the resulting solid material and its use as a medium for the oleaginous yeast Metschnikowia pulcherrima was explored. The cold press allowed the extraction of approximately 39% of the crude protein. Ball milling assisted with 5% Na_2_CO_3_ provided a drastic increase in the surface area and decrease in the particle size, albeit it did not significantly alter the structure in favor of enzymatic hydrolysis. A subsequent 0.5% NaOH pretreatment achieved enhanced fermentable sugar production with 45.6 g/L total sugars realized, notably 11-fold and 3.8-fold higher compared to untreated and mechanochemical-treated samples, respectively. M. pulcherrima efficiently metabolized all monosaccharides presented in supplemented ryegrass hydrolysates, yielding 0.20 (*Y*_m/m_) biomass with a 38.7% lipid content, similarly in a synthetic medium. Apart from being a lignocellulosic feedstock-based fermentable sugar production system serving the microbial lipid bioprocess, cold-press integrated mechanochemical biorefinery was shown to be a promising approach in the extraction of plant protein, while preventing robust separation operations before the severe chemical biorefinery stages of ryegrass.

## Graphical abstract

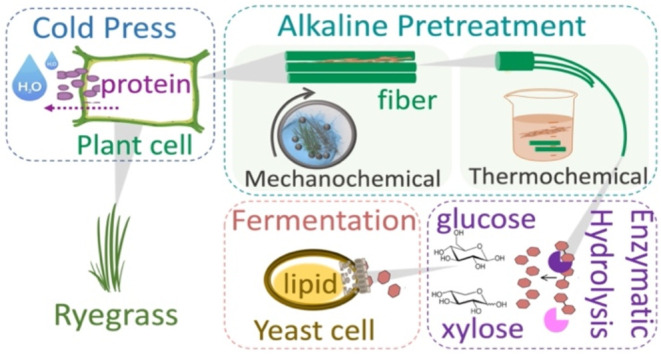

## 1 Introduction

Mechanochemical treatment has been widely used in the fractionation of lignocellulosic feedstocks, including ryegrass.^1,2^ Its mechanism relies on the depolymerization or breakage of linkages between the biomass components^3^ and has been used before chemical or biochemical treatments, leading to improved efficiency and economics of biomass transformation.^4^ Having the potential to be scaled up to produce more uniform products and to be conducted continuously makes it advantageous over chemical pretreatments.^5^ The mechanical deconstruction and extraction effect relies on applied pressure via frictional heat provided by kinetic energy without external heat^6^ and accelerates reaction rates.^7^ As materials are subjected to mechanical forces and chemical actions, mechanochemistry prevents high energy consumption to reduce particle size for the operation.^8^ Ball mill-integrated mechanical treatments primarily increase the specific surface area of plant fibers through crushing, fiber separation, and shearing force.^9^ Subsequently, the plant cell wall is destroyed by compressing the microfibrils. This results in the deconstruction of the fiber, leading to the partial breakage of the phenol-ether bond within the lignin molecule.^10^ Later, it reduces particle size by weakening the network of hydrogen bonds which hold the cellulose fibers together, so it leads to the amorphization of the crystalline structure.^11^ Thus, cellulose and hemicellulose become much more accessible to enzymes or chemical catalysts.^12^

Notably, the carbon source is the most significant medium component required for high microbial biomass production, which constitutes the major cost among medium supplements, accounting for around 38–73% of the production cost for fermentation processes.^13^ Therefore, alkaline, mineral salts, ammonia, and ionic liquids were previously applied in the mechanochemical pretreatment of feedstocks, including perennial ryegrass, due to their delignification feature.^2,5,14–19^ Particularly, alkaline pretreatment was reported to be effective not only due to its delignification effect but also due to its removal of hemicellulose fraction, thereby affecting the digestion of cellulose.^20^ Moreover, it was shown to overcome undesired side compound formation such as furfural, 5-hydroxymethylfurfural (HMF), acetic acid, and phenolics, which are major limiting factors inhibiting microbial growth.^21^ On the other hand, it causes the extraction of the protein fraction, presenting between 6 and 16% of ryegrass.^22,23^ Oleaginous yeast can metabolize C5 and C6 sugars;^24^ however, hydrolysates will still need to be supplemented to give optimal levels of other essential elements such as nitrogen. It is especially important to obtain the correct balance of N, as it is vital for the fermentation to be completed in N-limited conditions to induce lipid accumulation.^25^

Various pretreatment methods were studied to make use of the carbohydrate fraction of grasses as a second-generation feedstock, particularly cellulose, due to the metabolization limitation of the hexose hemicellulose hydrolysates (i.e., xylose, arabinose, and galactose).^19^ However, there are few studies on the integration of protein extraction into the pretreatment process, i.e., ammonia treatment of elephant grass^26^ and switchgrass.^27^ Moreover, the majority of studies on sustainable plant protein focus on the extraction of mainly the protein fraction of various crops^28^ and plants, including ryegrass.^29^ Despite requiring a low concentration of nitrogen supplementation to design an oleaginous yeast medium, alkaline pretreatment results in washing out the protein of grass from the system. Thus, this indicates the need to develop a biorefinery process that adopts a more circular approach. Cold press, not only having operational simplicity and low energy consumption features, but also preserving the functional properties of proteins, makes it favorable.^30^ Therefore, the effect of cold press on the protein extraction of Italian ryegrass (Lolium multiflorum ssp. *Italicum*), coupled with mechano- and thermochemical pretreatments, allowing the prediction of structural and enzymatic digestibility changes of lignocellulose at the lab scale, has been studied. This work further investigated the formulation of Italian ryegrass hydrolysates as an oleaginous yeast medium to establish a simple and cost-effective approach.

## 2 Materials and Methods

### 2.1 Materials

The grass type was Italian ryegrass (Lolium multiflorum ssp. *italicum*), sown and harvested in 2023. It was obtained in oven-dried form from Harper Adams University, U.K., and stored at room temperature. The ryegrass sample particle size was not larger than 1.4 mm. The Cellic CTec3 enzyme was used for depolymerization (Univar Solutions LLC, Illinois). The oleaginous yeast Metschnikowia pulcherrima NCYC4331 was used and deposited in the culture collection of the Chemical Engineering Department of the University of Bath.

### 2.2 Treatment

The cold press was performed with a domestic cold press machine (Nebula Grande Slow Juicer, NB-400) by using room temperature distilled water. The solid/liquid ratio was 1:39 (w/w), the soaking time was 14 min, and the processing time was 15 min, as determined by preliminary experiments. Afterward, the sample was centrifuged at 3000 rpm for 15 min, and the total nitrogen content of the liquid fraction was measured by TOC. The residue was collected and dried in an oven at 45 °C. Mechanochemical treatment (CBR) was applied in the planetary ball mill at 30 min of milling time with a 600 rpm milling speed and 1:30 residual to 5% (w/v) Na_2_CO_3_ solvent ratio (Fritsch, pulverizette 6), according to Olalere et al.^31^ Alkaline pretreatment of the mechanochemically treated sample (CBR) was done with 0.5% (w/v) NaOH, with a 5% (w/v) loading ratio. All experiments were run in a 250 mL bottle on a hot plate coupled with a thermocouple under vigorous mixing conditions at 75 °C for 1 h. At the end of the reaction, the mechanochemical- and alkaline-pretreated sample (CBAR) was cooled to room temperature, neutralized with 6 M HCl, and dried in a 105 °C oven overnight. The moisture content of the samples was measured according to Guler et al.^32^ Total nitrogen (TN) amounts were measured in the TOC-L instrument (Shimadzu) and converted to the protein amount by using a 6.25 factor. The treatments applied prior to enzymatic hydrolysis are summarized in Table 1.

**Table 1:** Applied Treatments to Ryegrass Prior to Enzymatic Hydrolysis^a^

	sample name	
treatment	CBR (Cold-pressed and ball-milled ryegrass)	CBAR (Cold-pressed, ball-milled, and alkaline-pretreated ryegrass)
cold press, 1:39(w/w) loading, water, 14 min soaking, 15 min press	+	+
mechanochemical treatment,1:30 (w/w) loading, 5% Na_2_CO_3_, 600 rpm ball milling speed, 30 min	+	+
alkaline Pretreatment, 5% (w/v) loading, 0.5% NaOH, 75°C, 1 h	—	+

a +: applied, —: not applied.

### 2.3 Biomass Characterization

A Brunauer–Emmett–Teller (BET) instrument and a Fourier transform infrared (ATR-FT-IR) spectrophotometer were used to analyze the difference between the untreated and pretreated biomass. SEM images were obtained using a Hitachi SU3900 SEM operated at 10 kV. All samples were sputter-coated with gold before analysis, and SEM images were obtained at different magnifications. Infrared spectra of the samples were recorded with a Bruker FT-IR INVENIO spectrometer. The samples were analyzed in the absorption band mode in the range of 400–4000 cm^–1^. The sample particle size was adjusted by sieving under 250 μm before measurements. Thermogravimetric analysis (TGA) of the 25 mg sample was performed as follows: a heating rate of 10 °C/min in the temperature range from 50 to 900 °C with nitrogen flow at a rate of 50 mL/min in duplicates using a Netzsch STA 449 F1 Thermogravimetric Analyzer. The surface area, pore volume, and pore size were measured by a Micromeritics 3 Flex instrument under nitrogen conditions.

### 2.4 Enzymatic Hydrolysis

Enzymatic hydrolysis was conducted in 50 mM sodium citrate buffer at pH 4.8 and 50 °C in an incubator for 72 h. The ryegrass loading ratio was 8% (w/v), and the total reaction volume was 60 mL. The reaction was conducted in a 250 mL shake flask in 150 rpm shaking conditions in an incubator. The activity of cellulase was determined according to the NREL protocol.^33^ Cellulase activity was measured at 326.8 FPU/mL and was used as 30 FPU/g of biomass. Enzymatic hydrolysis was performed according to Guler et al.^32^ Fermentable sugar was defined as the sum of glucose, xylose, arabinose, and cellobiose.

### 2.5 Ryegrass Hydrolysate Composition

The presence of carbohydrates, organic acids, and other grass depolymerization compounds in the ryegrass hydrolysate was screened in the initial and postincubation stages, both in the growth kinetic and the cell biomass experiments. Defrosted samples were centrifuged at 13,500 rpm for 5 min before analysis. Samples were screened for glucose, xylose, arabinose, cellobiose, formic, acetic, levulinic acid, HMF, and furfural by Agilent 1260 HPLC equipped with RI Detector, Aminex Bio-Rad HPX-87H column 300 × 7.8 mm, according to Guler et al.^32^ Total organic carbon (TOC) and total nitrogen (TN) amounts were measured with a TOC-L instrument (Shimadzu). Anion and cation contents were measured by ion chromatography (Metrohm).

### 2.6 Growth Kinetics

Ryegrass hydrolysate stored at −20 °C was defrosted at room temperature before medium adjustments. Ryegrass hydrolysate media were designed by adding chloramphenicol, tetracycline, and ammonium sulfate and applying pasteurization at 60 °C for 30 min, 80 °C for 10 min, sterilization at 120 °C for 15 min, and filtration by sterile 0.2 μm PVDF microfilter, separately. Ammonium sulfate was added to adjust the TOC to TN ratio to above 20, which supports lipid production.^25^ Stock solutions of (NH_4_)_2_SO_4_, chloramphenicol, and tetracycline were filtered through a sterile 0.2 μm PVDF microfilter before adding 100 and 15 mg/mL of stock solutions to the medium. Ammonium sulfate was used as a control for each medium in which antibiotics were not added. Standard nitrogen limited broth (NLB) medium (7 g/L KH_2_PO_4_, 2 g/L (NH_4_)_2_SO_4_, 1 g/L NaHPO_4_, 1 g/L yeast extract, 1.5 g/L MgSO_4_.7H_2_O, 80 g/L glucose, pH 5) and antibiotic-added NLB media were prepared by adding 15 μL/mL tetracycline or chloramphenicol.

The growth kinetic experiments were performed in a 96-well plate with 250 μL of adjusted ryegrass hydrolysate medium in a plate reader incubator (Thermoscientific Multiscan FC). M. pulcherrima NCYC4331 culture was activated according to Guler et al.^32^ A 24 h active culture was inoculated with a 2.5% (v/v) ratio into adjusted media; each medium was run in triplicate and with a noninoculated culture, which was used as a blank for calculations. Samples were incubated at 25 °C with low-speed shaking for 72 h, and the OD_600_ was measured every 30 min. The noninoculated absorbance values were subtracted from the absorbance values of the inoculated medium. OD_600_ data was first manipulated^34^ and then processed in Python.^35^ Growth kinetics wer calculated using RStudio software, and the data were fitted to a standard form of the logistic equation, which is a differential equation model.^36^ Contamination was checked by streaking samples on iron chloride-added malt extract agar (15 g/L agar, 30 g/L malt extract, 5 g/L soy peptone, 0.02 mg/L FeCl_3_) after 3 days of incubation at 25 °C.^37,38^ The redness of the colonies was evaluated to differentiate M. pulcherrima NCYC4331 from the fungal and yeast colonies. This was done before the inoculation of the culture and after 72 h of incubation of the media.

### 2.7 Cell Biomass and Lipid Production

The 24 h active culture was used for the inoculation of M. pulcherrima into the medium, as discussed in section 2.6. Citrate buffer was used in the enzymatic hydrolysis stage, which was later transferred into the ryegrass hydrolysate medium. Previously, 1 log of pH change resulted in a decrease in fermentation kinetics of M. pulcherrima, indicating the role of the buffer of the medium.^39^ Therefore, phosphate buffer used in the standard NLB medium formulation was replaced with citrate buffer to compare the effect of the buffer type on M. pulcherrima growth and lipid production at 25 °C. Active M. pulcherrima culture was inoculated at a 2.5% (v/v) ratio into the adjusted medium; each medium was run in triplicate. A noninoculated sample was used as a blank for experiments. Experiments were conducted in 20 mL of adjusted medium in a 100 mL shake flask and incubated at 25 °C at 200 rpm for 140 h. Measurement of dry cell weight (DCW) was done according to Abeln et al.,^39^ and cell lipid extraction was done according to Abeln et al.^37^ Contamination was checked, as discussed in Section 2.6. The formulations of the modified NLB and ryegrass hydrolysate media are listed in Table S1.

Samples were analyzed for fermentable sugar depletion, dry cell weight, lipid, and other metabolites produced at the end of the 140 h incubation. The amounts of glucose, xylose, arabinose, cellobiose, formic, acetic, and levulinic acid in the hydrolysate were detected, as described in Section 2.5. Total carbon and nitrogen measurements were conducted in a Shimadzu TOC-L instrument. Lipid extraction was done according to Abeln et al.,^37^ and esterification of the lipids and screening for the fatty acid methyl ester (FAME) profile was done according to ISO 12966-4.^40^ Samples were separated using an SH-Stabilwax-DA (30mt, 0.25 mm ID, 0.25 μm film) column in GC (Shimadzu Nexis GC-2030) equipped with a BID detector. Helium was used as the carrier gas at a flow rate of 1.5 mL/min. The initial temperature was set to 40 °C, held for 1 min, increased to 200 °C at a rate of 20 °C/min, and held for 60 min.

### 2.8 Statistical Analysis

Experimental data were analyzed with the Tukey comparison test in IBM SPSS Statistics software.

## 3 Results and Discussion

### 3.1 Effect of Pretreatment on Depolymerization of Ryegrass

Cold press resulted in the extraction of 54.8 mg g^–1^ crude protein from ryegrass, representing approximately 39% protein content of Italian ryegrass according to the literature.^41^ The total mass loss was found to be 45.3%; therefore, water extractives could have been removed within 15 min of pressing. Solely, sodium carbonate-assisted ball milling has been previously reported to extract the protein fraction of the moor grass as 63%.^31^ Adding the cold press step prior to mechanochemical treatment recovered a substantial amount of water extractives, mainly protein, that make up the majority of the extracted portion, which otherwise requires complex separation from the black liquor.^42^ Techno-economic analyses of microbial lipid production from waste were conducted for the scale-up of culturing M. pulcherrima,^43,44^ and its industrial feasibility in large-scale applications from brewery waste was demonstrated by integrating and optimizing multiple pilot-scale unit operations.^45^ These studies indicated that predominantly, feedstock price, lipid yield, and selling the whole yeast cell have a major impact on the economic viability of the process, while an integrated brewery waste biorefinery was shown to be industrially feasible.^45^ In this respect, extracting protein in a high yield and keeping the overall energy consumption low throughout the mechanical and thermochemical treatment could be potential challenges for the scale-up of the protein extraction integrated biorefinery process.

The intensity change of the peak absorbance was mainly observed in wavenumbers 1031, 1383, 1590, 1635, 2851, 2916, and 3331 cm^–1^, and all decreased with applied treatments to ryegrass (Figure 1a). The sharp peaks at 1031, 2851, and 2916 cm^–1^ dropped significantly. This indicates compositional differences between the untreated and treated ryegrass samples. The peak at 1031 cm^–1^ is related to C–O–C–O–C stretching bonds of cellulose; the peak at 1383 cm^–1^ shows C–H deformation in the cellulose and hemicellulose structure; the peaks at 1590 and 1695 cm^–1^ regions show aromatic ring stretching related to lignin removal; the peaks at 2851 and 2916 cm^–1^ show −OCH_3_ and C–H stretching related to aromatic groups and rupture of cellulose, respectively; and the peak at 3331 cm^–1^ shows O–H stretching related to rupture of cellulose and water content.^46^ Applying alkaline pretreatment in addition to mechanochemical treatment did not change the intensity of the many presented peaks in CBR. Previously, a peak at 1635 cm^–1^ was associated with the aromatic skeleton vibration of lignin, which decreased for the CBR and CBAR samples. This shows lignin removal or alteration.^47^ The solubilization of lignin resulted in similar changes to the FT-IR spectrum of Na_2_CO_3_-pretreated Miscanthus and switchgrass.^48^ Alkaline pretreatment specifically disrupts the lignin-cellulose/hemicellulose ester linkage and acetyl groups of hemicellulose. The intensity of the band at 1383 cm^–1^ decreased in both CBR and CBAR samples, possibly due to the removal of hemicellulose by Na_2_CO_3_ and NaOH.

**Figure 1:**
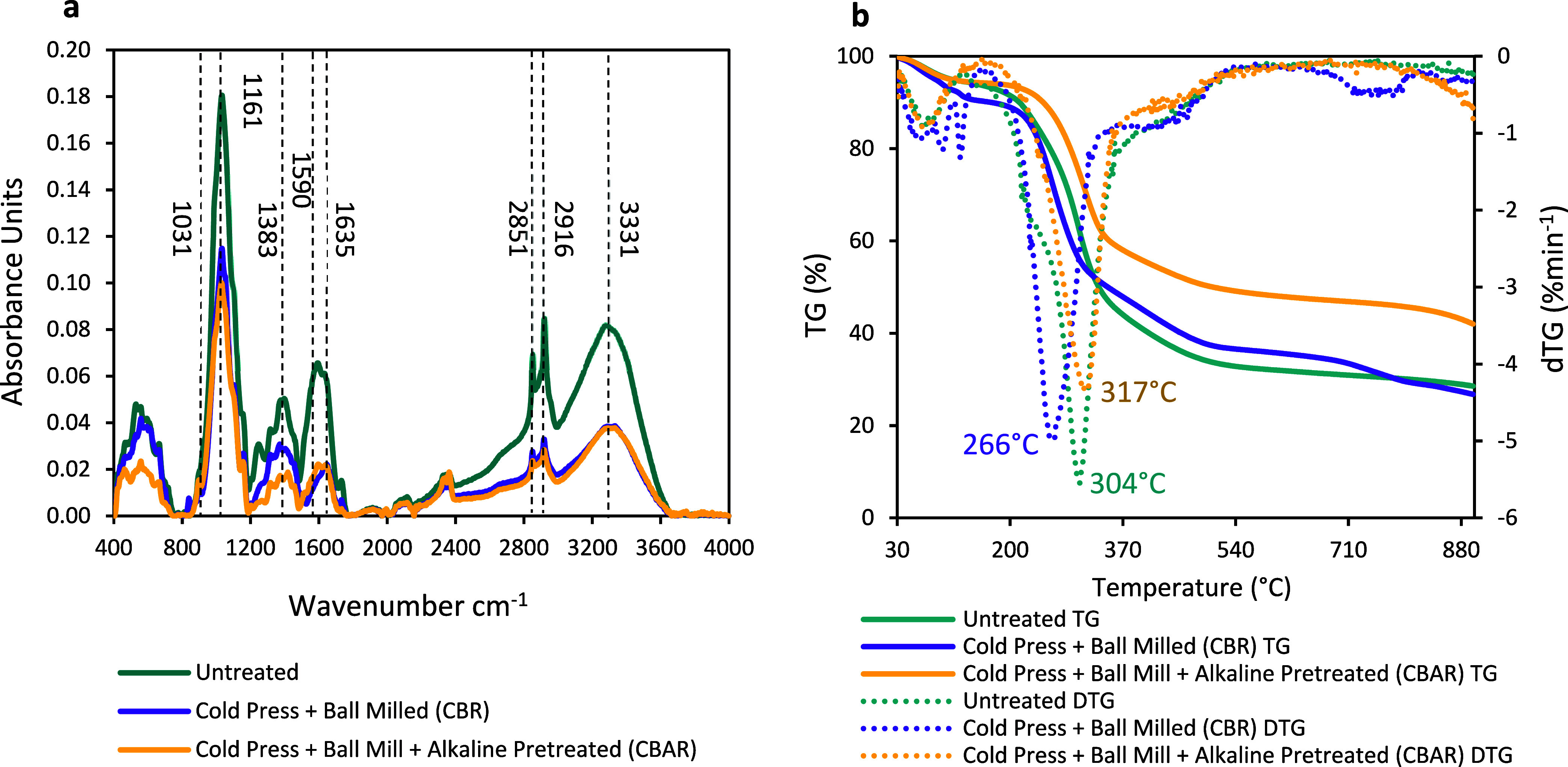
(a) Fourier transform infrared spectra (baseline-corrected) and (b) thermogravimetric (TG) and derivative thermogravimetric (dTG) curves of untreated, CBR, and CBAR samples.

Thermogravimetric (TG) and derivative thermogravimetric (dTG) curves represent the decomposition of the ryegrass samples to understand the changes in their chemical properties after treatment (Figure 1b). Previously, mass change during lignocellulosic biomass decomposition was divided into three stages: mainly, the range of 180–350 °C is attributed to the degradation of components of structural carbohydrates and lignin; above 350 °C is attributed to the degradation of volatile products such as phenolics, alcohols, and aldehyde acids along with the formation of gaseous products form after degradation of lignin, and under 180 °C is associated with water removal.^46,49^ Substantial weight loss occurred at 304 °C, 266 and 317 °C, for untreated, CBR, and CBAR samples, and the residual weight ratios were 28.6%, 26.8%, and 42.0%, respectively. Maximum weight losses were observed between 140 and 580 °C, with an amount of 62.1% and maximum at 304 °C for the untreated sample; maximum at 180–360 °C with an amount of 41.6% and maximum at 266 °C for the CBR sample. Also, another mass loss of 13.1% between 360 °C-620 °C was observed. The CBAR sample showed maximum weight loss at 160 °C-580 °C with an amount of 45.9% and maximum at 317 °C.

The CBR and CBAR samples have a lower dTG value than untreated ryegrass in the region between 180 and 360 °C, indicating less mass release, which implies a chemical compositional change with applied treatments. Untreated and CBAR showed similar behavior in the cellulose degradation zone, which indicates that their compositions can be similar in terms of cellulose percentage. A significant shoulder was observed in the dTG curve for the untreated sample at 266 °C, in contrast to CBR and CBAR. Shoulders at around 217 and 235 °C were previously related to hemicellulose-releasing temperature ranges. Besides, the FT-IR spectra of CBR and CBAR in the hemicellulose removal zone support this conclusion.

Since the required bond dissociation energy for the degradation of lignin is higher due to its phenylpropane units, C=C bonds, and aromatic nature, it decomposes in a wide temperature range and slowly compared to carbohydrates. The region above 350 °C is where fixed carbon releases, and it was found to be highly correlated with the lignin content previously.^50^ Low dTG is associated with the release of less volatile compounds, in which the mass change of CBAR above 350 °C was lower than untreated and CBR. This can be explained by its low lignin content.^51^ The CBR sample showed the highest mass loss above 350 °C; this can be related to its lignin ratio. The increased proportion of the composition due to cold pressing and ball milling might have removed the water extractives and hemicellulose. The residual mass of CBAR was higher than that of the untreated and CBR. This can be attributed to salt formation at the HCl neutralization stage and subsequently a lack of washing, resulting in ash residue.^52^ Although the CBR showed a TGA curve similar to that of the CBAR, its mass loss started at a low temperature and extended to a higher temperature, resulting in a higher mass loss. Overall, thermogravimetric analysis suggests an alteration in the ryegrass chemical composition.

Untreated ryegrass showed a compact, fibrous, and smooth surface morphology, while the CBR sample had a disorganized, less fibrous, and porous structure with cracks, as seen in the SEM images (Figure 2a–f). CBAR also showed a less fibrous structure along with increased fiber surface deformation compared to CBR (Figure 2g–i). Deformation of the rigid surface of the ryegrass fiber was increased by applied treatments, which led to an increase in the availability of the carbohydrate fraction, thereby increasing the yield of enzymatic hydrolysis (Figure 2c,f,i). This can be explained by the fact that NaOH pretreatment applied at mild conditions affected the physical structure much more harshly than Na_2_CO_3_-assisted ball mill treatment, which was previously reported to increase the solubilization of lignin and hemicellulose by Ginni et al.^53^

**Figure 2:**
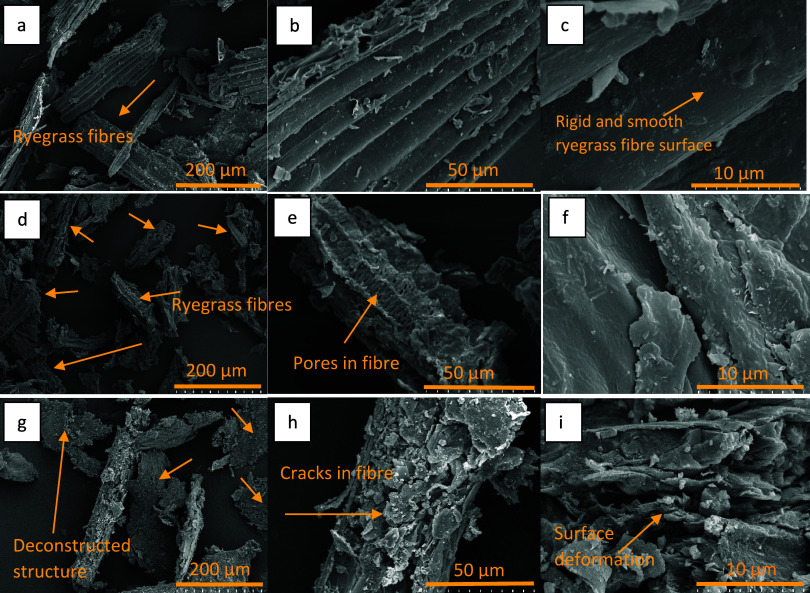
Scanning electron microscope image of untreated (a: 200X, b: 1000X, c: 5000X); CBR (d: 200X, e: 1000X, f: 5000X); CBAR (g: 200X, h: 1000X, i: 5000X) Italian ryegrass.

Brunauer–Emmett–Teller (BET) and Langmuir surface areas, pore and nanoparticle sizes, and pore volumes derived from the t-plots, which are standard values for the characterization of porous materials, were used to evaluate the physical changes of mechanochemically and NaOH-pretreated ryegrass. The BET surface area increased significantly from 0.36 to 1.94 m^2^/g after cold press and ball milling with Na_2_CO_3_, albeit decreased to 0.54 m^2^/g with the alkaline pretreatment (Table 2). However, the sample, after being pretreated with an alkaline solution, resulted in a decrease in the surface area, absorption volume of pores, and pore size. The same trend was observed for the Langmuir surface area and nanoparticle size. Formation of the new surface through rupture of the van der Waals, covalent bonds, and the defects of the crystal structure, thereby increasing the reactivity of the components, is defined as the result of mechanical stress.^54^ Additionally, wet ball milling has been reported to break the phenol-ether bonds within the lignin molecule, leading to increased hydrophilicity and reactivity of the lignin.^10^ In contrast to surface area results, the subsequently applied dilute NaOH pretreatment under mild conditions induced significant compositional changes, particularly through the removal of lignin and hemicellulose fractions, which revealed the modified decomposition characteristics by FT-IR and TGA analysis.

**Table 2:** Physical Features and Production of Fermentable Sugar from Untreated and Treated Italian Ryegrass

	sample name		
features	untreated ryegrass	cold-pressed + ball mill-treated ryegrass (CBR)	cold-pressed + ball-milled + alkaline-pretreated ryegrass (CBAR)
Surface Area			
BET surface area, m^2^/g	0.36	1.94	0.54
Langmuir surface area, m^2^/g	0.63	31.96	3.64
Pore Volume			
single-point adsorption total pore volume of pores less than 40.3122 nm in diameter, cm^3^/g	3.87 × 10^–4^	54.25 × 10^–4^	8.69 × 10^–4^
*t*-plot micropore volume, cm^3^/g	1.20 × 10^–4^	0.21 × 10^–4^	0.18 × 10^–4^
Pore Size			
adsorption average pore diameter (4 V/A by BET), nm	4.25	11.18	6.40
Nanoparticle Size			
average Particle Size, nm	16,490.29	3090.06	11,050.94
Produced Total Fermentable Sugar, g/L	4.08	12.75	45.60

The fermentable sugar concentration produced from the CBR sample was 12.75 g/L, while it increased 3.57 times after additional pretreatment with NaOH. There was no direct correlation between the BET surface area and enzymatic hydrolysis rate, in contrast to Wiman et al.^55^ Similarly, Zhou et al.^9^ reported that ball milling increased the specific surface area of the fiber, albeit it hardly improved the enzymatic hydrolysis rate. This was also explained by the decrease in the crystallinity and polymerization of cellulose that enhances enzymatic hydrolysis depending on the ball milling time.^9^ Besides, dilute alkali-assisted ball milling coupled with hydrothermal pretreatment at mild conditions was reported to enhance the enzymatic saccharification efficiency of grass.^15^ Yielding a higher fermentable sugar concentration with the CBAR sample indicates that not only do microstructural changes affect enzymatic digestibility, but also favorable chemical composition shifts are crucial. This is because alkaline pretreatment not only removes or alters the structure of lignin and hemicellulose but also increases the surface area and pore volume. This later leads to an increase in the enzymatic hydrolysis rate.^56^ This indicates that without applying severe alkaline conditions, the concentration of produced fermentable sugar can effectively be increased to 45.6 g/L, compared to 12.75 g/L that was otherwise yielded. Besides, it does not require detoxification as an additional unit operation.^19^ The SEM and surface analysis showed that Na_2_CO_3_-assisted ball milling crushed the ryegrass fibers into small and thin particles and increased the pore volume of the fiber, albeit it did not lead to significant depolymerization of the carbohydrate fraction. It can be concluded that enhancing the enzymatic accessibility of lignocellulosic biomass through mechanochemical treatment is highly dependent on the assisted chemical characteristics rather than the applied physical forces.

### 3.2 Growth Kinetics of M. pulcherrima

The growth rates (*r*) and growth kinetic curves were calculated on NLB and ryegrass hydrolysate media after 72 h of incubation at 25 °C (Figures 3 and 4). There were no significant differences in the growth rates and the maximum OD_600_ values between chloramphenicol and tetracycline-added NLB media compared to the control (standard NLB) (*p* > 0.05). The same growth trend was observed for antibiotic-added ryegrass hydrolysate medium. However, the growth rates were lower than NLB, possibly because the initial fermentable sugar concentration was 34.4 g/L lower than the NLB medium. Maximum OD_600_ values reached 1.0 in ryegrass hydrolysate for both antibiotic-added and control (microfiltered) ryegrass media (Figure 4). A lower growth rate was observed in both ammonium sulfate-supplemented and unsupplemented autoclaved ryegrass hydrolysate with a maximum OD_600_ value of 0.08 (Figure 4c,d). Ammonium sulfate supplementation did not cause a significant change in the growth rate and maximum OD_600_ values for all supplemented media except the chloramphenicol-added medium.

**Figure 3:**
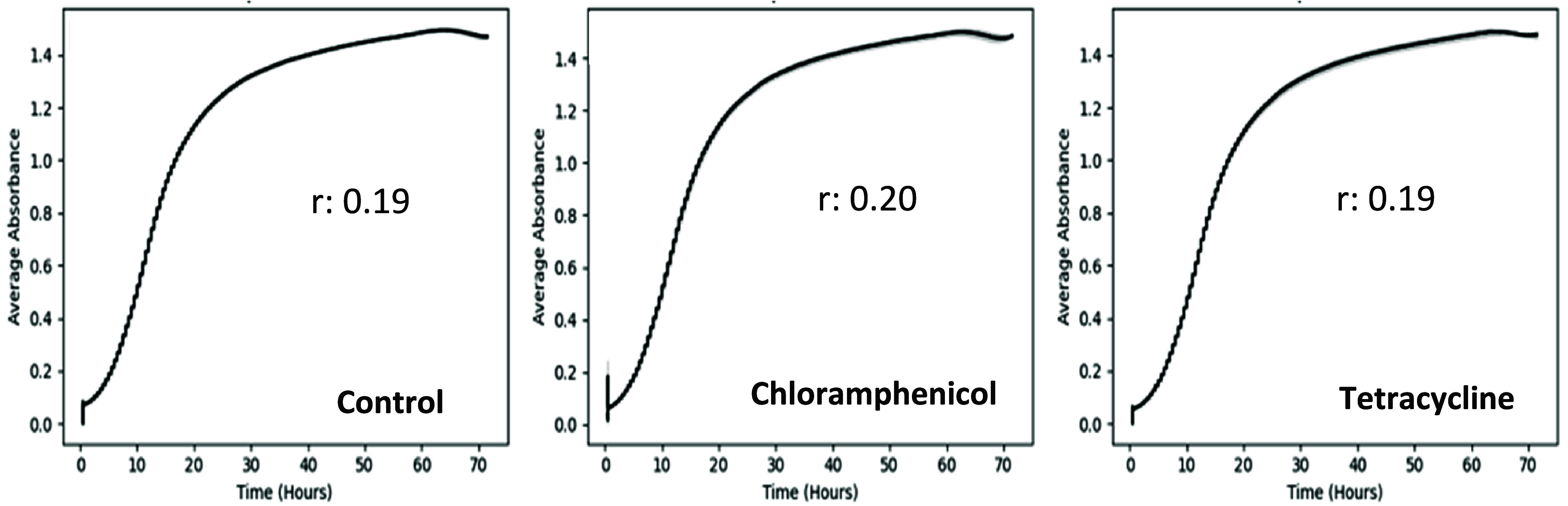
Growth kinetics and growth rate (*r*) of M. pulcherrima NCYC4331 on autoclaved standard NLB (control), chloramphenicol, and tetracycline-added NLB media at 25 °C after 72 h incubation. The *X*-axis represents the average absorbance at OD_600_, and the *Y*-axis represents the incubation time (hours).

**Figure 4:**
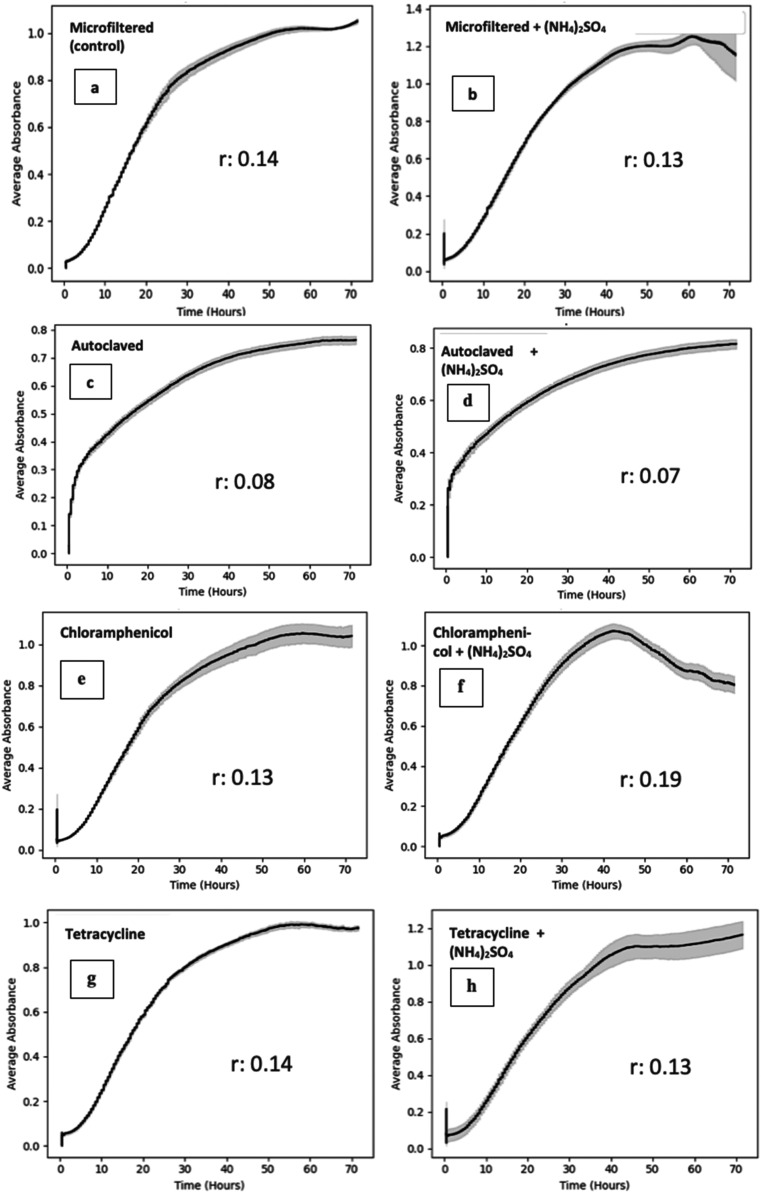
Growth kinetics and growth rate of M. pulcherrima NCYC4331 on adjusted Italian ryegrass hydrolysate at 25 °C after 72 h incubation. (a) Microfiltered (control); (b) microfiltered + ammonium sulfate; (c) autoclaved; (d) autoclaved + ammonium sulfate; (e) chloramphenicol; (f) chloramphenicol + ammonium sulfate; (g) tetracycline; and (h) tetracycline + ammonium sulfate. The *y*-axis represents the average absorbance at OD_600_, and the *x*-axis represents the incubation time (hours).

### 3.3 Cell Biomass and Lipid Production

The standard NLB medium showed a lower dry cell weight and lipid concentration compared to all other formulated versions. Besides, a higher pH drop was observed for this medium (Figure 5). The produced dry cell weight from citrate buffer and yeast extract formulated NLB medium was the highest, with 15.48 ± 2.16 g/L and a 50.9% lipid ratio; similar results were obtained for citrate buffer, yeast extract formulated, and tetracycline-added medium with 14.58 ± 2.09 g/L and a 50.4% lipid ratio. However, ammonium sulfate-supplemented modified NLB medium resulted in a significant decrease in the dry cell weight (7.87 ± 1.45 g/L) and lipid (34.7%) (*p* < 0.05).

**Figure 5:**
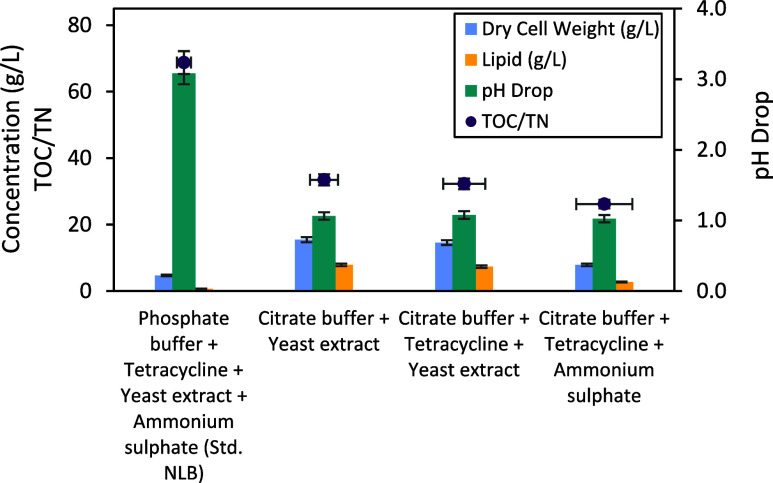
Effects of buffer, nitrogen source type, and antibiotic on dry cell weight and lipid concentration in the standard and modified NLB media at 25 °C after 140 h incubation (hold value for glucose concentration: 80 g/L).

The pH buffering capacity of the medium was increased by adding citrate buffer. This was previously explained by the medium’s acidification due to the release of protons from the consumption of ammonium salts or the accumulation of organic acids during yeast cultivation.^57^ Formulating a medium by adding yeast extract instead of solely using ammonium sulfate increased 1.85 times the dry cell weight amount, indicating that yeast extract not only provides nitrogen but also provides higher nutritional value than previously shown for M. pulcherrima.^39^ The modified NLB medium formulated with yeast extract substantially improved the dry cell weight and lipid accumulation at 25 °C and showed a higher concentration compared to all other formulated versions.

The partial removal of soluble proteins from ryegrass in the cold press and pretreatment steps suggests that additional nitrogen supplementation was required for maintaining sufficient nitrogen, correlating with improved yeast biomass yields. Therefore, the nitrogen concentration of the ryegrass hydrolysates was adjusted by adding ammonium sulfate to keep the medium cost low. In this way, it was aimed to bring the TOC/TN ratio of CBAR hydrolysates from 136.59 to 20.95, which is a favorable condition for lipid secretion. After 140h of fermentation in the ryegrass hydrolysate medium at 25 °C, the pH dropped from 5.19 to 4.67. M. pulcherrima consumed approximately all C_6_ and C_5_ monosaccharides present in the ryegrass hydrolysate (Table 3). With the consumption of 44.69 ± 0.01 g/L fermentable sugar, 8.78 ± 1.52 g/L dry cell weight was produced with a 38.7% lipid ratio. The major cation consumed was ammonium, with 33.12% of the initial amount among the cations, and chloride consumed 45.9% of its initial amount (Table 3). The highest unsaturated fatty acid ratio was observed when the medium was formulated with citrate buffer and ammonium sulfate, similar to the ryegrass hydrolysate medium formulation, indicating that ammonium sulfate can be preferred as a low-cost nitrogen source without resulting in a drastic change in the FAME profile (Table 4).

**Table 3:** Cation, Anion, and Monosaccharide Profile of Ammonium Sulfate-Supplemented Ryegrass Hydrolysate Medium and Consumption^a^

Compound	Initial Amount	Consumed Amount
Cation		
ammonium, g/L	9.78 ± 0.16	3.24 ± 0.04
calcium, ppm	302.85 ± 5.16	101.15 ± 5.73
lithium, ppm	trace amount	trace amount
magnesium, ppm	36.40 ± 3.25	33.42 ± 2.04
potassium, ppm	153.20 ± 4.67	44.40 ± 0.57
sodium, ppm	TA	NA
Anion		
bromide, ppm	4.87 ± 0.81	4.87 ± 0.81
chloride, g/L	9.28 ± 0.11	4.26 ± 0.12
fluoride, ppm	146.90 ± 2.42	133.03 ± 2.42
phosphate, ppm	181.30 ± 6.51	121.05 ± 10.68
sulfate, g/L	2.25 ± 0.01	1.14 ± 0.01
Total fermentable sugar	45.60 ± 0.66	44.69 ± 0.01
glucose, g/L	32.25 ± 0.24	31.93 ± 0.24
xylose, g/L	11.83 ± 0.07	11.27 ± 0.23
arabinose, g/L	1.51 ± 0.00	1.50 ± 0.00

a Displayed is the mean ± SD of the two replicates. TA: trace amount, NA: not available. Incubation time: 140h; scale: shake flask; incubation temp.: 25 °C.

**Table 4:** FAME Concentration of Lipids Obtained in the Standard and Modified NLB Media, and Ammonium Sulfate-Supplemented Ryegrass Hydrolysates (GH)

	concentration (mg FAME/mg lipid)			
medium composition	C16:0	C16:1	C18:1	C18:2
ryegrass hydrolysate (GH) + ammonium sulfate + tetracycline	0.074 ± 0.02	0.027 ± 0.01	0.092 ± 0.01	ND
glucose + phosphate buffer + yeast extract + ammonium sulfate + tetracycline^a^	0.065 ± 0.00	0.058 ± 0.01	0.103 ± 0.00	0.034 ± 0.01
glucose + citrate buffer + yeast extract + tetracycline[Table-fn t4fn2]	0.111 ± 0.02	0.026 ± 0.01	0.143 ± 0.00	0.031 ± 0.00
glucose + citrate buffer + ammonium sulfate + tetracycline[Table-fn t4fn2]	0.097 ± 0.00	0.022 ± 0.01	0.118 ± 0.01	0.017 ± 0.01

a Standard NLB.

b,c Modified NLB. ND: not detected. Displayed is the mean ± SD of the two replicates. Incubation time: 140 h; scale: shake flask; incubation temp.: 25 °C.

## 4 Conclusions

Cold pressing using water, a simple unit operation, allowed the extraction of 54.8 mg g^–1^ crude protein from Italian ryegrass. Ball milling with Na_2_CO_3_ did not increase enzymatic hydrolysis significantly compared with the untreated sample. Its subsequent combination with a dilute NaOH pretreatment yielded 45.60 ± 0.66 g/L fermentable sugar, mainly consisting of 32.25 ± 0.24 g/L glucose, 11.83 ± 0.07 g/L xylose, and 1.51 ± 0.00 g/L arabinose. Citrate buffer in the grass hydrolysate formulation supported yeast growth better than phosphate buffer at 25 °C fermentation temperature. Supplementing hydrolysates with yeast extract as a nitrogen source resulted in approximately two times higher dry cell weight than ammonium sulfate. M. pulcherrima metabolized all glucose, xylose, and arabinose presented in the supplemented ryegrass hydrolysate medium, yielding 8.78 g/L yeast biomass with a 38.7% lipid content. The higher biomass was associated with the buffering capacity and nitrogen type of the oleaginous yeast medium, which led to a biomass production yield of 0.20, consisting of a lipid profile of mainly oleic, palmitic, and palmitoleic acids. The addition of antibiotics, both in the enzymatic hydrolysis and fermentation stages, did not have a significant impact on M. pulcherrima growth kinetics and biomass yield while ensuring uncontaminated fermentation, irrespective of the utilized antibiotic type. Overall, the findings suggest that integrating the cold press step prior to chemical treatments recovered a substantial amount of protein. This shows the nutritional potential of Italian ryegrass as a plant protein source, in addition to being a sustainable fermentable sugar replacement feedstock.

## Supplementary Material



## Notes

Additional experimental details regarding the formulation of Italian ryegrass hydrolysates and modified nitrogen limited broth (NLB) media (Table S1) (PDF)

## Abbreviations

NLB: nitrogen limited broth
TOC: total organic carbon
TN: total nitrogen
NREL: National Renewable Energy Laboratory
FPU: filter paper unit
PVDF: poly­(vinylidene fluoride) filtration membrane
HPLC: high-performance liquid chromatography
GC: gas chromatography
FT-IR: Fourier transform infrared
BET: Brunauer–Emmett–Teller
SEM: scanning electron microscope
Y_m/m_: biomass production yield
DCW: dry cell weight
OD: optical density
FAME: fatty acid methyl ester
HMF: hydroxymethylfurfural
TGA: thermogravimetric analysis
dTG: derivative thermogravimetric
GH: grass hydrolysates
CBR: cold-pressed and ball-milled-treated ryegrass
CBAR: cold-pressed, ball-milled, and alkaline-pretreated ryegrass
