# Supplementary material for: Integrated Chemical and Biochemical Treatments to Produce Protein and Microbial Lipid Food Ingredients from Ryegrass
Source: ACS Sustain Chem Eng. 2025 Jun 13;13(25):9588–98. doi: 10.1021/acssuschemeng.5c02288 (PMC12216236; doi:10.1021/acssuschemeng.5c02288)
Supplement: Supplementary file 1 [file sc5c02288_si_001.pdf]

Supporting Information

Integrated Chemical and Biochemical Treatments to Produce Protein and Microbial Lipid Food Ingredients from Ryegrass

Fatma Guler, Yubin Ding, Hannah S. Leese, Bernardo Castro-Dominguez, and Christopher J. Chuck*

Department of Chemical Engineering, University of Bath, Claverton Down, Bath BA2 7AY, United Kingdom

Table of Contents

Table S1

Table S1. Formulation of Italian ryegrass hydrolysates and modified Nitrogen Limited Broth (NLB) media

Medium Name	Tetracycline, µg/mL	Arabinose, g/L	Xylose, g/L	Glucose, g/L	Yeast Extract, g/L	Ammonium Sulphate	$MgSO_4.7H_2O$, g/L	KH_2PO_4, g/L	$NaHPO_4$, g/L	Grass Hydrolysate	Sodium Citrate Buffer, mM
**Ryegrass Hydrolysate[a] (GH)-Tetracycline + $(NH_4)_2SO_4$**	15	1.5	11	32	-	+	-	-	-	+	50
**NLB-Tetracycline**	15	-	-	80	1	+	1.5	7	2	-	-
**NLB-Citrate buffer[c]**		-	-	80	1	-	1.5	-	-	-	50
**NLB-Citrate buffer + Tetracycline[d]**	15	-	-	80	1	-	1.5	-	-	-	50
**NLB- Citrate buffer + Tetracycline + $(NH_4)_2SO_4$[e]**	15	-	-	80	-	+	1.5	-	-	-	50

[a] Ryegrass Hydrolysate (GH) composition used in the shake flask experiment is given in Table 2. [b] Standard NLB [c, d, e] Modified NLB. + : Contain, - : Not contain.
